# Decision-making of the benthic diatom *Seminavis robusta* searching for inorganic nutrients and pheromones

**DOI:** 10.1038/s41396-018-0299-2

**Published:** 2018-10-09

**Authors:** Karen Grace V. Bondoc, Christine Lembke, Stefan N. Lang, Sebastian Germerodt, Stefan Schuster, Wim Vyverman, Georg Pohnert

**Affiliations:** 1 Institute for Inorganic and Analytical Chemistry, Bioorganic Analytics, Friedrich-Schiller-Universität Jena, Lessingstrasse 8, D-07743 Jena, Germany; 2 Max Planck Institute for Chemical Ecology, Hans-Knöll-Str. 8, D-07745 Jena, Germany; 3 Department of Bioinformatics, Friedrich-Schiller-Universität Jena, Ernst-Abbe-Platz 2, D-07743 Jena, Germany; 4 Laboratory of Protistology and Aquatic Ecology, Department of Biology, University Gent, Krijgslaan 281 S8, 9000 Gent, Belgium; 5 Present Address: Department of Marine and Coastal Sciences, Rutgers University, New Brunswick, NJ 08901-8521 USA; 6 Present Address: Department of Experimental Limnology, Leibniz-Institute of Freshwater Ecology and Inland Fisheries, Alte Fischerhütte 2, D-16775 Stechlin, Germany

**Keywords:** Microbial biooceanography, Microbial ecology, Microbial ecology

## Abstract

Microorganisms encounter a diversity of chemical stimuli that trigger individual responses and influence population dynamics. However, microbial behavior under the influence of different incentives and microbial decision-making is poorly understood. Benthic marine diatoms that react to sexual attractants as well as to nutrient gradients face such multiple constraints. Here, we document and model behavioral complexity and context-sensitive responses of these motile unicellular algae to sex pheromones and the nutrient silicate. Throughout the life cycle of the model diatom *Seminavis robusta* nutrient-starved cells localize sources of silicate by combined chemokinetic and chemotactic motility. However, with an increasing need for sex to restore the initial cell size, a change in behavior favoring the attraction-pheromone-guided search for a mating partner takes place. When sex becomes inevitable to prevent cell death, safeguard mechanisms are abandoned, and cells prioritize the search for mating partners. Such selection processes help to explain biofilm organization and to understand species interactions in complex communities.

## Introduction

Diatoms are a species-rich group of silicifying microalgae responsible for about 20% of global carbon fixation [1]. As major primary producers in marine and freshwater ecosystems, they shape the aquatic and global carbon and silicon biogeochemical cycles [2]. Motile pennate diatoms often dominate the soft sediment substrata of aquatic habitats, forming biofilms that can contribute substantially to ecosystem productivity and provide important ecosystem services such as sediment stabilization [3]. These phototrophic biofilms typically represent a dynamic, spatially heterogeneous environment. They are characterized by strong and fluctuating gradients in physical conditions and resource availability. Consequently, it can be hypothesized that diatoms in biofilms have evolved multiple strategies to maximize their fitness under such variable constraints [4]. Motile species, in particular, can rapidly respond to external cues, possibly contributing to patchiness within biofilms [5, 6]. Directional responses in many raphid pennate diatoms are enabled by a characteristic forward and backward movement that is mediated by the excretion of adhesive extracellular polymeric substances [7]. Additionally, cells can undergo turning movements using pseudopod-like structures that temporarily attach to the substrate [8].

The directed movement of gametangial cells is essential in the mating behavior of many motile diatoms once they have entered the sexual phase that is defined in their life history as follows [9]. During cell division of diatoms, each daughter cell inherits one part of the parental biomineralized cell wall (theca) and forms a new theca through precipitation of incorporated dissolved silicate (dSi). Since the formation of new thecae is occurring within the parental cell, a gradual decrease in mean cell size of the offspring is observed over time. When a specific sexual size threshold is reached, cells of opposite mating types can pair and form gametes through meiosis which fuse, resulting in auxospore formation, and subsequently in the development of large initial cells that can again undergo repeated mitotic cell divisions [9–11]. If mating is not possible (e.g., due to the absence of a mating partner), cells will die once below a critical minimal cell size [10, 12]. Pennate diatoms employ elaborate pheromone systems to synchronize sexuality and to attract mating partners [11, 13]. Recently, a first diatom pheromone was identified as the l-proline-derived diketopiperazine (in the following abbreviated as diproline) in *Seminavis robusta* [14]. It mediates the chemoattraction of cells of the mating type^+^ (MT^+^) to the corresponding diproline-producing mating type^-^ (MT^-^) cells. Analysis of the attraction of *S. robusta* towards diproline sources revealed that the cells use a chemotactic and chemokinetic movement to approach a pheromone source [15]. Diproline production and perception capabilities are synchronized by sex-inducing pheromones (SIP) that are released by the respective mating types once they reach the sexual size threshold [14, 16].

Motility aids not only in the location of mating partners, it can also direct towards or away from other environmental cues. A significant body of research has demonstrated the role of motility in the response of biofilm-forming diatoms to photoperiod, light quality, and tidal cycles [17, 18]. Even nutrients can direct diatoms as recently evidenced by the search of silicic acid [19]. This mineralic acid is a common limiting factor for diatom growth as silicate is the major constituent of their intricate biomineralized cell walls [10, 20]. While the general effect of nutrient limitation on diatom growth and metabolism has been intensively studied, little is known about their behavioral response towards gradients of such resources. In fact, *S. robusta* is attracted to point sources of dSi with a similar behavior that follows chemotactic and chemokinetic patterns as the search for pheromones [19].

In this contribution, we explore how diatoms respond to multiple chemical cues. We examine how responses towards dSi and the attraction pheromone are manifested under different environmental and physiological constraints to elucidate the process of microbial decision-making. Indeed, diatoms flexibly adjust their behavior according to their cell size, sexual priming, and nutrient availability within short periods of time. We explore biofilm dynamics through mathematical modeling of the interaction of cell density and resource availability. This work provides new insights into biofilm organization and function and suggests that searching behavior depends on lifecycle state, mating partner availability, as well as on the strength and nature of competing attractors.

## Materials and methods

### Diatom strains and microscopy

We used the *Seminavis robusta* strains 85A (mating type MT^+^) and 84A (mating type MT^−^), which are maintained in the BCCM/DCG diatom culture collection at Ghent University (http://bccm.belspo.be/about-us/bccm-dcg). The strains used had different cell sizes in apical length below and above the sexual size threshold [14]. We classified them as small- (24–27 µm), medium- (~ 40 µm), and large-sized cells (> 50 µm). Additional information is given in the supporting material.

### Attraction and choice assays with dSi- and l-diproline-loaded beads

l-diproline-loaded beads were prepared modified after Gillard et al. [14] by using solid phase extraction material from HLB cartridges (Oasis®, Waters, Eschborn, Germany). We chose a final concentration of 4 nmol diproline per mg beads, which is within the bioactive concentration range of the pheromone [14] and was previously used for movement pattern analysis [15]. dSi beads were prepared according to Bondoc et al. [19], wherein 1.4 nmol dSi was loaded per bead. This optimal concentration elicited the highest accumulation of cells around the bead [19]. Aliquots of < 30 dSi beads or 5 µg of l-diproline-loaded beads were added to each well, and the movement of the cells was monitored for 10 min. For choice assays, both beads were applied simultaneously. Attraction assays were modified from previous studies as described in the supporting material [14, 15]. The observation radius for subsequent analysis was set to 115 µm from the edge of the bead to account for size differences of the beads.

### Video and statistical analyses

Movies for counting and tracking were re-processed from 100 fps to 1 fps using VirtualDub (http://virtualdub.org/) and Fiji (http://fiji.sc/). The open-source tracking plug-in, TrackMate (http://fiji.sc/TrackMate) [21] was used in automatic mode for all experimental sets. Automatic tracking parameters of the simple LAP tracker were first optimized on an individual movie before using it for the whole experimental data set. All data analyses were done under the open-source statistical program R v. 3.3.1 [22] with the packages ggplot2 [23] for plotting, nlme [24] for linear mixed effects (LME) modeling, and mgcv [25] for general additive mixed modeling (GAMM). Specific model formulations are given in Table S1, and the results of all statistical analysis are summarized (Supplementary Tables S3–S8).

### Mathematical modeling of decision-making responses

Here we utilize a mathematical model to describe the switch between mitotic growth and sex for varying combinations of cell density (as a proxy for SIP availability) and resources (as a proxy for dSi concentration). We investigate the relationship of the critical cell size threshold, to the long-term mean growth rate in cell lineages arising from single individuals of both sexes. All cells are assumed to use the same strategy. The mean long-term growth rate of the lineages is then used as a fitness proxy for the decision strategy. Defining two alternative optimality criteria, the model helps us to identify regions of (critical) cell sizes, where either mitosis or meiosis (or both) should be preferred, depending on the environmental conditions. Model parameters are described in Supplementary Table S2. The model takes into account (i) the changes in cell size of an individual (for *S. robusta* the starting value of 75 μm (*size*_*init*_) derived from microscopic investigations is assumed), (ii) the probability for successful meiotic reproduction (*r*_*enc*_), and (iii) the fitness cost ratio of growth and sex, i.e., mitosis and meiosis. The estimated growth rate (*gr*) of all offspring arising from a single initial cell is calculated as:

$$gr = \frac{{\ln \left( N \right)}}{{g_{max} \ast t}}_{d_{mitosis}}$$

where *N* is the estimated total number of offspring a single initial cell may generate within a fixed number of generations (*g*_*max*_) by choosing growth or sex given by a threshold size, *size*_*crit*_, below which meiosis rather than mitosis is performed. *t*_*dmitosis*_ refers to the time required for a mitotic division. The simulation runs over a total number of *g*_*max*_ = 4500 generations to estimate stable solutions.

To determine the numbers of offspring with specific cell sizes and their total number, *N*, time series (*n*) were created using the following recursion function:

$$n\left( {0,c_{max}} \right) = 1,n\left( {0,c} \right) = 0\,{\mathrm{for}}\,{\mathrm{any}}\,c\, < \,c_{max}$$

$$n\left( {g,\,c} \right) = n\left( {g - 1,\,c + 1} \right) + n\left( {g - 1,\,c} \right) \cdot$$

*n(g, c)* is the number of individuals of the current size class in the current generation, *n*(*g−1, c+1*) and *n*(*g−1, c*) are the total numbers of the next largest size class and of the current size class, respectively, in the previous generation. Each diatom cell has a theoretical initial size (*size*_*init*_) of 75 µm corresponding to the size class *c*_*max*_. During mitotic division, a decrease in size, shrinkage of 0.7 µm is assumed, corresponding to the doubled cell wall width. Diatoms with a cell size below a minimum size threshold (*size*_*min*_) of 10 µm, corresponding to *c*_*min*_, were assumed not to be viable and were excluded from calculations. Numerical values for cell sizes were estimated by light microscopy.

To simulate meiotic reproduction, a fraction of individuals *n*(*g, c*)*a(*g*) is temporarily removed from the time series of their respective size class to account for the time required for mating. The resulting offspring is later added on to the highest size class. Thereby, *a(g)* is the probability for successful meiotic division within the current generation. This includes the probability of detecting other individuals through pheromone mediation and the movement towards a competent potential individual:

$$a\left( g \right) = b\left( {g - 1} \right) \ast r_{enc}$$

Here, *b(g−1)* represents the fraction of sexual cells in the previous generation (cells below a specific sexual size threshold, *size*_*crit*_). The rate at which a single cell can encounter another potential competent individual within its detection radius is represented by *r*_*enc*_. This probability is scaled from d^−1^ to generations^−1^. Meiosis is assumed to generate two new individuals that are added to the series on the highest size class and to remove the two small mating cells for further calculations. The time span required for meiosis (*t*_*meiosis*_) is taken into account. This simulates the size-recovering effect of meiotic division. The series is generated for *g*_*max*_ generations and all size classes. We assumed the time for meiosis (*t*_meiosis_) and time for mitosis (*t*_*dmitosis*_) to depend on growth conditions. Since mitotic growth is only possible upon nutrient acquisition, the ratio of mitosis to meiosis can serve as proxy for the availability of resources (i.e. dSi).

The optimality criterion (i) is to maximize *N* by choosing mitotic growth or sex depending on cell size. As an alternative optimality criterion (ii), a linear combination of *N* and the estimated average cell size is maximized. The latter is calculated as $\mathop {\sum }\limits_{g,c} n\left( {g,c} \right) \ast size\left( c \right)$. The coefficients in the linear combination are chosen such that both of its terms are normalized to be within 0 and 1. Model validations and further information can be found in the supporting material.

## Results

### Diatoms switch between nutrient and mate-searching behavior depending on their needs

Given the constraints of the need for nutrients and the finding of sexual partners, diatoms are challenged to locate different chemical signals in a patchy environment. We hypothesize that diatoms can prioritize between different stimuli depending on their nutrient requirements and readiness for sex. To test this hypothesis, we investigated the behavioral responses towards pheromones and dSi. From our previous observations [15, 19], we were able to choose relevant concentrations and timescales for the attraction experiments to make the comparison in this study robust. We experimentally focus on the three distinct phases in the *S. robusta* life cycle (i) large-sized cells > 50 µm that have just undergone restoration of their cell size by meiosis, (ii) medium-sized cells ~ 40 µm that have crossed the sexual size threshold but are still capable of mitotic division, and (iii) small-sized cells < 27 µm that are in the need for sexual reproduction since a further reduction in size would result in cell death. A clear priority (i.e., preferred accumulation) for dSi of large-sized cells was observed, which are attracted to dSi once starved (Supplementary Figure S1A) but do not respond to pheromones [14]. The accumulation of medium and small-sized cells to dSi-loaded beads was comparable to the response of large-sized cells to this resource, albeit the latter required a longer starvation time to exhibit attraction behavior (Supplementary Figure S1B).

**Fig. 1:**
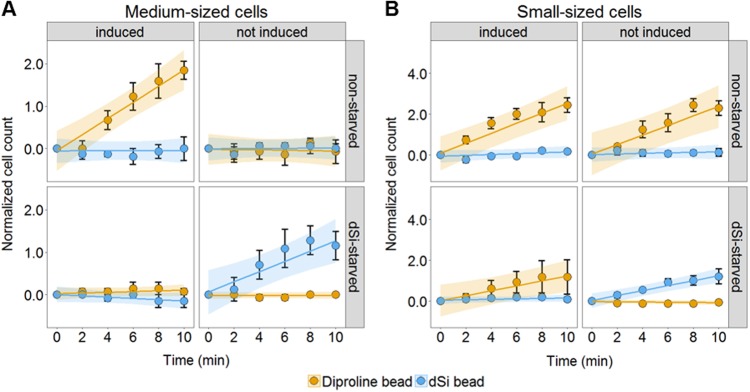
Cell size dependent behavioral switch of *S. robusta*. The combined effects of sex induction and dSi starvation were determined on medium-sized (~ 40 µm) and small-sized (24–27 µm) *S. robusta*. All data points are presented as mean ± SEM of 3 movies except dSi-starved induced and not induced (*n*_(movies)_ = 6) as cell responses showed variability between sets. The overlaid shaded area shows the linear mixed effects modeling (LME) with pair-wise Tukey’s honest significance difference (HSD) fit with 95% confidence intervals, detailed statistical analysis can be found in Table S3. **A** Medium-sized cells were only attracted to diproline when grown with dSi and upon induction (*p* < 0.001 compared with all treatments). dSi starvation and non-induction render the cells to be attracted to dSi beads (*p* < 0.01 compared with all treatments). **B** Non-starved, small-sized cells were attracted to diproline, even without induction and at the same intensity as induced cells (*p* = 1 compared to the non-starved and induced treatment). However, these self-inducing cells were not attracted to the pheromone when starved, and it was only through induction that starved cells regained attraction to diproline (*p* = 0.18). Only small cells that are both not induced and starved were attracted to dSi (*p* < 0.02 for all comparisons)

To further explore behavior under multiple constraints, we tested all possible combinations of starvation and induction in small and medium-sized sexual cells (Fig. 1). In experiments where diproline beads were applied to medium-sized mating type^+^ cells, the attraction was only observed when cells were both nutrient replete and sexually induced by SIP. However, under dSi limitation, no response to diproline was observed even when SIP was present (Fig. 1A, orange entries). dSi starvation thus interferes with mate-searching behavior. On the other hand, the response to dSi beads was dependent on the sexual induction. The attraction of medium-sized cells to dSi beads was only observed when they were starved and not induced with SIP (Fig. 1A, blue entries). Readiness for sex thus suppresses dSi orientation.

Small-sized cells have a more pronounced response to diproline. If grown under dSi replete conditions, accumulation around diproline-loaded beads was evident even when cells were not exposed to SIP (Fig. 1B). In contrast to the medium-sized cells, the attraction of small cells towards diproline took also place when dSi-starved. However, this behavior was only observed when cells were sexually induced by addition of SIP to the medium. Only under the combination of the adverse conditions dSi starvation and absence of SIP, no response to diproline was observed. It should be noted that small amounts of dSi (~ 50 µM) were carried over during SIP exposure, as this had to be done through the addition of spent medium from exponentially growing cultures of the opposite mating type. Cells slowed down but remained motile, therefore we can conclude that no substantial interference due to dSi in the assay can be expected (Supplementary Figure S2).

### Diatoms exhibit a context-sensitive selection between resource (dSi) and mating partner location

**Fig. 2:**
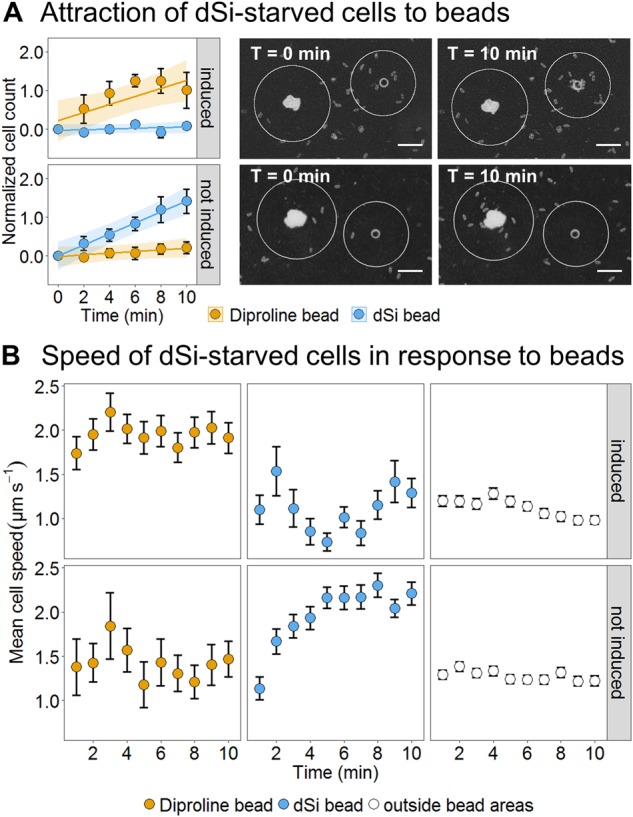
Choice experiments (dSi vs. diproline). Choice experiments were performed using self-inducing and dSi-starved small cells. **A** The left panel shows normalized cell counts from bins (depicted as white circles in the pictures right) having a size of 115 µm from the edge of the bead. Induced cells accumulated only on diproline beads over time (orange circles and upper panels *p* = 0.0259). On the other hand, the not induced cells showed preference only to dSi beads (blue circles, lower panels *p* = 0.0003). Data points are presented as mean ± SEM of 3 videos from independent treatments. Pheromone-loaded beads are placed right in the assays. The overlaid shaded area shows the LME model fit with 95% confidence intervals. Detailed statistical analysis can be found in Table S4. Scale bar = 100 µm. **B** The cell speed averaged over intervals of 1 min for 10 min were taken from automatically tracked cells in five independent movies (*n*_(cells/movie)_ = 80–100). The track data was divided into three groups: the areas around the two beads and the area outside the beads. Induced cells showed a consistent higher mean speed when cells are under the influence of diproline gradients compared to dSi and outside the beads (LME with Tukey’s HSD, *p* < 0.001 for both). In contrast, non-induced cells that accumulated around dSi beads increase speed over time, whereas, diproline did not induce any change in speed (*p* < 0.001). Data points are presented as mean speed ± SEM of 5 videos from independent treatments. Detailed statistical analysis can be found in Table S5

To investigate potential selection behavior, we performed choice experiments with dSi-starved small cells that were either SIP induced or non-induced. If both dSi and diproline-loaded beads were applied simultaneously to each well, starved cells were attracted towards diproline beads when induced and to dSi beads when not induced (Figs. 1B and 2A, Movies 1 and 2). Indicative of an orthokinetic response, cells moved significantly faster when they were around the stimuli that they were attracted to, increasing their speed two-fold (Fig. 2B) [15]. Within the 10 min observation period, no switch towards mate-searching could be observed in starved and non-induced cells. These critically small, self-inducing cells chose dSi over diproline sources when they were starved and resource acquisition is thus preferred over mating even in this scenario of extreme pressure to gain cells of maximum size in the population.

### Diatom decision-making explained by mathematical modeling

**Fig. 3:**
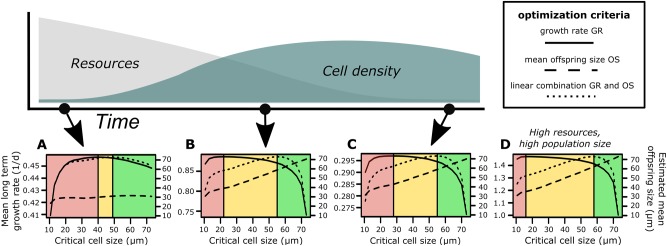
Numerical modeling on diatom decision-making. The mathematical model investigates the optimal choice of cells between mitotic division and sex depending on nutrient resources and cell density. The panel above illustrates the development of a natural population or lab culture and the consequent consumption of resources. The subplots **A–C** represent the modelled choices of the cells at distinct conditions encountered during this process. Subplot **D** is an extra scenario wherein a dense population experiences a high influx of nutrients. Modeled subplots of other conditions can be found in Supplementary Figure S3. Vertical lines on subplots **A–D** represent cell size thresholds at which a switch between optimal modes of reproduction, i.e. mitotic divisions and sex occurs. The first threshold (transition red-to-yellow) appears if the optimization criterion is solely the expected growth rate (solid line). By adding a second optimization criterion, the estimated offspring size (dashed line), in linear combination with the estimated growth rate (dotted line) a second threshold was determined (transition yellow-to-green). Individuals of size classes within the red area should choose meiosis as the primary mode of reproduction, while individuals of size classes within the green area should always choose mitosis. The yellow area represents size classes of individuals that should choose mitosis according to the optimal expected number of offspring but may choose meiosis to additionally preserve mean cell size. The plots show that the optimal switching points significantly alter along typical population dynamics and resource availability. Note that the mean long-term growth rates given are a fitness proxy and not equivalent to growth rates in cultures

To explain the observed decision-making of *S. robusta* within evolutionary constraints, we developed a mathematical model describing expected dynamics of a population where individuals would preferably switch from asexual reproduction to sex if their cell size drops below a critical size (*size*_*crit*_). Mitotic growth results in size reduction of one daughter cell while pairing of two cells will recover the maximal species-specific cell size. By testing different encounter probabilities between individual cells (as proxy for SIP availability) and altering the weighting regimes of the ratio of the mitotic and meiotic generation time, a broad variety of environmental conditions can be modeled, including low and high cell densities, high and low resource availabilities (as proxy for dSi concentration), and combinations of both parameters. In Fig. 3, ecologically-motivated scenarios are assigned to the sketch of a typical development of a population starting with high resources and low cell densities and ending with low resources and high densities. We modeled three different scenarios that would be relevant in such a growing population—low densities and high resource availability (Fig. 3A), mean densities and mean resource availability (Fig. 3B), and high densities low resource availability (Fig. 3C). In addition, we modeled a situation with high densities and high resource availability that would occur after nutrient influx into a dense population (Fig. 3D). Modeling results from a broader range of parameters are found in the Supporting Material Figure S3.

The model revealed a significant shift of the optimal *size*_*crit*_ for the transition to preferred sexual pairing, depending on the environmental conditions. This parameter is also dependent on the consideration if solely the expected growth rate or a combination of expected growth rate and size preservation is used as the optimization criterion (Fig. 3). In our experiment, this is translated to a priority for diproline when cells are below the critical size. The two vertical lines in each of the subplots A-D represent *size*_*crit*_, where individuals should switch their mode of reproduction according to (i) optimal expected growth rate solely (e.g., grow fast) or (ii) in linear combination with optimal preservation of mean cell size (e.g., ‘grow-fast-and-stay-big’). The ranges for each strategy alter with external conditions. Green areas represent those size classes where diatom cells should optimally choose mitotic growth and ignore SIP signals according to both optimization criteria (i) and (ii). In this state, optimizing vegetative growth rate has higher importance than conserving the future offspring size. This area declines with decreasing resources and increasing cell density. Red areas represent diatom size classes where individuals should be susceptible to SIPs and prefer the meiotic mode of reproduction, according to optimization criterion (i). Otherwise, the expected future growth rate drops and the mean size of the offspring would shrink, as well as the probability of successful mate finding and size restoration. Yellow areas represent size classes of diatoms where cells should choose mitosis according to optimization criterion (i), but meiosis according to optimization criterion (ii). This means that within the yellow range, individuals can choose sex to enlarge cell size, but may also choose asexual reproduction to optimize the expected number of offspring. Note that scales and values of the estimated mean long-term growth rates and cell sizes in Fig. 3 result from idealized model conditions dependent on the specific parameter settings (Table S2) and may differ numerically from natural scenarios, while the qualitative behavior remains the same. Thus the specific values are not equivalent to and may not be directly compared to real-world data but reflect general reproductive strategies.

At low cell densities, the probability of finding a partner for sexual pairing is low (Fig. 3A). Consequently, the mean offspring size that is dependent on the encounter rates remains small. Although growth conditions are optimal due to high resource availability, a switch to the meiotic strategy should be considered by medium-sized individuals (40–49 μm). With the imminent risk of becoming smaller during mitotic divisions and not finding an appropriate partner due to low density, individuals should switch to meiosis even if growth conditions appear optimal at a *size*_*crit*_ of 40 μm. At mean cell densities and mean resource availabilities (Fig. 3B), both the probability for a successful meiotic reproduction event and the resource availability for successful mitotic growth is high. Therefore, the yellow area depicting the size range where cells can choose between meiosis and mitosis is wider (22–55 μm). At this condition and at higher cell densities the estimated mean offspring size is rather dependent on the term “critical cell size” that reflects the sexual strategy (Fig. 3B–D). Under high densities and high resource availabilities, this yellow area is even more extensive (17–58 μm, Fig. 3D). Hence, small- and medium-sized cells can stick to mitosis and risk a decrease in the size of their offspring. At high cell densities and low resource availabilities (Fig. 3C), the yellow area becomes smaller and the red area extends to 28 μm cell size. Under this condition, cells can only choose between growth and sex when they are in the size range between 28 and 55 μm. For all subplots, except for Fig. 3A, cell size is highly correlated (*r* = 1) with the linear combination of growth rate and future offspring cell size.

## Discussion

Microorganisms contribute to the micro-scale community structure by selecting suitable microhabitats and by exploiting ephemeral and spatially varying nutrient sources. Additionally, the location of mating partners or avoidance of competitors can be guided by the perception of chemical stimuli [5, 6]. Theoretical considerations suggest that the presence of several currencies (e.g., nutrients, sex) generally results in the evolution of higher lifetime reproductive success through partial circumvention of such trade-offs. Experimental validation, especially for microorganisms, is, however, highly challenging [26]. We introduce the benthic diatom *S. robusta* as a model for such behavioral studies in microorganisms. Its response towards nutrients and pheromones can be independently triggered and easily monitored in biofilms under different limitation scenarios. The underlying migration towards chemical attractants is an important control mechanism for the allocation of nutrients and mating partners [27]. Motility of diatoms is a cost-efficient mechanism to forage for nutrients and search mating partners in a patchy environment, as only ~ 0.001% of the predicted power of the cell is consumed for moving at a speed of 10 µm s^−1^ [28].

In this study, movement patterns of vegetative and sexual cells of different sizes and degrees of sex induction were monitored under the influence of the macronutrient dSi, and the attraction pheromone diproline. A 10 min observation time was selected based on previous experiments. Longer observation times are not required since cells either stop moving in dSi gradients due to the successful localization of local maxima [15, 19] or move away due to unsuccessful pairing in diproline gradients [15]. We have previously shown that small dSi-starved *S. robusta* cells exhibit an active searching behavior to gradients of this mineral acid [19]. In the current study, this type of behavior was consistently observed in medium- and large-sized cells (Fig. 1 and Supplementary Figure S1), indicating a universal response towards this stimulus throughout the life cycle. However, vegetative cells required a longer starvation time than small cells below the SST, probably due to physical constraints by having a decreased cellular surface area: volume ratio, thereby increasing nutrient uptake demand [29]. Additionally, they possess a thicker boundary layer, a nutrient-depleted area around the cell that inhibits transport of nutrients through diffusion from the bulk concentration in the environment [30]. Together, these two factors would require large-sized cells to substantially deplete their internal dSi pool, for cells to efficiently uptake dSi through diffusion and active foraging.

The searching behavior within a gradient of the pheromone diproline is strongly dependent on cell size and priming by SIP. Large cells above the sexual size threshold do not respond to diproline even under the influence of SIP, while cells that crossed the threshold are attracted to it [14]. *In silico* comparison of the two independent data sets led to the speculation that *S. robusta* has a more efficient behavioral adaptation to the pheromone gradient as compared to dSi [15]. In sexual cells just below the sexual size threshold, only under favorable conditions (nutrient availability and SIP priming), a search behavior for mating partners is initiated. This behavior changes when small cells near the minimal critical size are considered. These cells would die if further mitotic cell divisions would reduce their size and no size-restoration by sex would occur [31]. Accordingly, under nutrient replete conditions, small cells do not require induction by SIP to orient towards the attraction pheromone diproline, thereby exhibiting a self-priming behavior. The observed increased readiness for mating behavior might explain why reduced cell size is correlated with a higher reproductive success in mating *S. robusta* [32]. The self-induction mechanism represents a breach in the highly orchestrated synchronization of sex in diatoms and might be considered as an emergency mechanism to prime sexuality in the face of cell death.

In the case where *S. robusta* faces more than one constraint, again a shift in the responsive behavior is observed. Under dSi starvation, self-induction of the diproline-searching behavior of small cells is not initiated. This finding is in accordance with observations that cell proliferation only occurs when dSi is available [33, 34]. Guidance to a mating partner is inefficient under these conditions, as no resource for the formation of the novel cell wall is available. Instead, priority for dSi acquisition would be a logical consequence. This priority is also supported in the choice experiment where dSi-starved, not induced small cells still responded to dSi beads and did not switch to diproline within a 10 min observation time.

It becomes evident that successful pairing of diatoms is affected by the interacting factors of the probability of cell-to-cell encounter (high density of a population would lead to high encounter rates and hence low cost of sex) and the availability of sufficient resources (nutrients and energy) for completing auxosporulation [35]. To explain the decision-making observed in *S. robusta*, we applied a mathematical model that calculates the switch between mitotic growth and sex based on partner and resource availabilities. The model predications support the experimentally-observed context dependent preference for mitotic growth or meiotic reproduction. The fact that large cells solely select mitotic divisions is confirmed under all tested conditions (Fig. 3A–D, green area). The observation that the frequency of sexual pairing increases as cells become smaller [14, 32], has also been confirmed in our model (Fig. 3A–D, red area). The model includes a term for cell density that is directly determining encounter rates for mating. This term is equivalent to the presence (high cell densities) or absence (low cell densities) of SIP in our experimental setup, where partner availability was mimicked by SIP addition.

In accordance with the modeled conditions of high resource availability and high cell density (i.e., SIP availability), cells can select sexuality even if they just crossed the sexual size threshold as represented by the large yellow area in Fig. 3D. The experimental finding that for small cells under very favorable conditions no induction with SIP is required (self-priming in Fig. 2B) is supported by the model (Fig. 3A), suggesting that even at low densities (i.e., the absence of SIP) sex is preferred. When there are enough cells for mating (mean to high cell density), cells tend to choose between mitotic growth and sex, under varying resource availability. SIP induction is required, and resources and critical size for meiosis are inversely proportional to each other. That is, at low resources, the critical size is higher. Our model results also verified the observed switch on our experimental data (Fig. 3C). Large cells would always choose vegetative reproduction, medium cells can either choose to grow (by foraging nutrients) or to mate (by finding a mating partner), while small cells would always choose to mate. Moreover, the experimentally-determined sexual size threshold of ~ 52 μm [14] is within the calculated critical sizes for choosing between mitosis and meiosis (yellow area) on three regimes (Fig. 3B–D). Under favorable conditions, wherein both nutrients and partners are readily available, cells of a broader size range can choose between vegetative growth and sex. However, the future offspring size should also be considered. In some diatom species, parental and offspring size is positively correlated [36, 37], as it was observed in the model. As such, there should be a preference to switch to sex as fast as possible to ensure a higher initial cell size.

Our model also shows ecologically-relevant scenarios that could occur in diatoms during patch colonization. As most benthic pennate diatoms are highly motile and thus capable of foraging for resources, their initial attraction towards a nutrient patch, particularly of dSi, reflects the model dynamics. Hence, a specific patch could have subpopulations differing in cell size distribution and sexual pressure, which would be dependent on their location and proximity to a nutrient source. Indeed, during bloom conditions of *Pseudo-nitzschia*, other sexually reproducing diatoms and subpopulations of different size classes were observed; only a fraction of the population proceeded with sex at a given nutrient regime [35, 38].

Cellular decision-making is known for several microorganisms but has till now been reported to occur at the transcriptomic and not at behavioral level. Under specific constraints, such as nutrient limitation or conspecific interaction, even a microorganism is challenged to choose a response from a plethora of possibilities including morphological, physiological, transcriptomic, or even behavioral adaptations [39]. The nutritional quality of the environment requires the cell to allocate resources between reproduction, especially when nutrients are abundant, and cellular maintenance when nutrients are limiting [40]. For example, in *Escherichia coli*, nutritional status can trigger cells to decide between cell growth or cell maintenance and survival through regulation of transcriptional resources such as promoters [40]. In microbial eukaryotes where reproduction can also be sexual, mating success can be dependent on the nutrient requirement of the cell. On a genetic level, such dependence has been reported in the yeast *Saccharomyces cerevisiae* where mating response pathways can only be induced by a hormone under nutrient repletion [41]. For the microalgae, *Chlamydomonas reinhardtii* and *Salpingoeca rosetta*, sex can be triggered by specific nutrient limitations. However, behavioral studies on these species are limited to assays with single stimuli [42, 43]. As such, we offer a remarkable system for behavioral studies of eukaryotic microbes that can link foraging and reproduction behavior with a direct response to sexual and nutritional stimuli separately and in combination. This system demonstrates that even in a time scale of seconds, decisions can be made by the unicellular organisms. The immediate response requires different reception processes for the respective stimuli and the ability to respond in a directed orientation. The short timescale for decision-making renders the involvement of signaling pathway crosstalk between dSi-sensing and mating on a transcriptome level highly unlikely. This non-genetic response represents thus an adaptive allocation of time, energy, and nutrients as competing functions that is manifested in the complex behavior of the microorganisms. Transcriptomic evidence for overlaying processes is however emerging. In the diatom *Pseudo-nitzschia multistriata* nutrient transporters and cyclins involved in sensing nutritional status were downregulated during sex [44]. Moreover, both dSi limitation and SIP exposure can arrest the cell cycle in the G1/S phase in *S. robusta* [16] and secondary messenger cyclic nucleotides (cAMP or cGMP) may play a role in both pheromone perception and motility [44]. Future research should thus focus on the regulatory signaling pathways within the diatom cell and how they interact to maximize survival and fitness in a heterogeneous environment and during interaction with other species.

Our work demonstrates that pennate diatoms can alter their searching behavior across their life cycle and shows their ability to respond to and exploit small-scale patchiness in nutrient resources and the presence of mating partners in their habitat. While these findings help to understand the ecological and evolutionary success of pennate diatoms, this study also shows that life-history variation in unicellular organisms contributes to the integration of behavior and physiology within the environmental and size-dependent contexts of selection. As such, this study opens a wide avenue for future work to identify the mechanisms underlying decision-making in microorganisms as well as better to understand the roles of life history and environmental patchiness in shaping microbial communities and their ecological function.

## Electronic supplementary material

Supplementary Material

Supplementary Movie1

Supplementary Movie2
