# Supplementary material for: Decision-making of the benthic diatom Seminavis robusta searching for inorganic nutrients and pheromones
Source: ISME J. 2018 Oct 9;13(2):537–46. doi: 10.1038/s41396-018-0299-2 (PMC6331598; doi:10.1038/s41396-018-0299-2)
Supplement: Supplementary file 1 — Supplementary Material [file 41396_2018_299_MOESM1_ESM.pdf]

Supplementary Information

Decision-making of the benthic diatom *Seminavis robusta* searching for inorganic nutrients and pheromones

Karen Grace V. Bondoc[1, 2, 5, #, *], Christine Lembke[1, 6, #], Stefan N. Lang[3], Sebastian Germerodt[3], Stefan Schuster[3], Wim Vyverman[4], Georg Pohnert[1, 2, *]

[1] Institute for Inorganic and Analytical Chemistry, Bioorganic Analytics, Friedrich-Schiller-Universität Jena, Lessingstrasse 8, D-07743 Jena, Germany

[2] Max Planck Institute for Chemical Ecology, Hans-Knöll-Str. 8, D-07745 Jena, Germany

[3] Department of Bioinformatics, Friedrich-Schiller-Universität Jena, Ernst-Abbe-Platz 2, D-07743, Jena, Germany

[4] Laboratory of Protistology and Aquatic Ecology, Department of Biology, University Gent, Krijgslaan 281 S8, 9000 Gent, Belgium

[5] Present address: Department of Marine and Coastal Sciences, Rutgers University, New Brunswick, NJ 08901-8521, USA

[6] Present address: Department of Experimental Limnology, Leibniz-Institute of Freshwater Ecology and Inland Fisheries, Alte Fischerhütte 2, D-16775 Stechlin, Germany

[#] These authors contributed equally to this work.

* Correspondence: karen.bondoc@gmail.com (K.G.V.B.); Georg.Pohnert@uni-jena.de (G.P.)

Supplementary Methods

Strains, maintenance of stock cultures, and microscopy

Stock cultures of large-sized cells of mating type MT^- (84A) were grown in buffered artificial seawater (ASW) modified after Maier and Calenberg [1] with concentrations of 621 µM $NaNO_3$ (VWR Chemicals, Leuven Belgium), 155 µM K_2HPO_4 (Roth, Karlsruhe, Germany) and 246 µM $Na_2SiO_3 \cdot 9\ H_2O$ (Sigma-Aldrich, Steinheim, Germany). Stock cultures of strains MT^+ (85A) and small-sized MT^- (84A) were grown in 33g/l Instant Ocean® Sea Salt (Aquarium Systems, Sarrebourg, France) dissolved in deionized water with f/2 supplements according to Guillard [2]. Experimental cultures were prepared by inoculating one ml aliquots of 7-day old stock cultures, which are dSi-limited (small and medium-sized cells are already limited in Si after the first 5 days of culturing. Large-sized after 7 days of culturing had 30% of the initial Si available. By starvation of these large cells for 7 additional days they became limited in Si as well [3] , into 2 ml of either fresh ASW or nutrient-depleted ASW in 12-well plates (Sarstedt, Nümbrecht, Germany). Changing the medium did not result in cellular stress as observed by microscopy. All cultures were grown at 18 °C in a 12:12 h light: dark regime with cool-white fluorescent lamps at approximately 35 µmol photons $m^{-2}\ s^{-1}$. For microscopic observations, photography and video recording an inverted Leica DM IL LED microscope (Heerbrugg, Switzerland) mounted with a Nikon DS-Fi2 CCD camera (Tokyo, Japan) recording 100 fps was used. Illumination occurred from below and was homogeneous in the observation range to exclude overlaying effects of phototaxis.

Attraction assays

Attraction assays were modified from previous studies [14, 15, 21]. Experimental cultures were grown for 2 days before cell-cycle synchronization by prolonging the dark period to 36 h [19]. This resulted in cell densities of 13,000-24,000 cells cm^{-2} for small-sized non-starved

cells, 11,000-16,000 cells cm^{-2} for medium-sized non-starved cells, 8,000-12,000 cells cm^{-2} for small-sized dSi-starved cells, and 4,000-8,000 cells cm^{-2} for medium-sized dSi-starved cells. For induction, 1 ml of SIP-containing medium stock (prepared from small-sized 84A (MT^{-}) cultures that were sterile-filtered during the late exponential growth phase with cell densities of 14,000-19,000 cells cm^{-2}) was added to each well of MT+ culture before illumination. The attraction and choice assays were carried out 6 and 8 hours after the onset of illumination for small- and medium-sized cells, respectively.

Attraction of large-sized cells to dSi-loaded beads

Batch cultures of large-sized cells (MT^{-}, strain 84A), as well as dSi and control beads, were prepared as described previously. Cells were starved for one week before exposing them to beads for 10 min, as shorter starvation period did not elicit any reaction. Photos were recorded every minute, but cell counts were only determined every 2 min on a 115 µm observation area from the edge of the bead. Three replicates per treatment were used. A linear mixed effects (LME) model coupled with the post-hoc Tukey's HSD was used to analyze the data (Supplementary Figure S1A, Supplementary Table S6). The degree of attraction of dSi over different cell sizes was compared through statistical analysis of count data (Supplementary Figure S2B, Supplementary Table S7).

Motility of 85A small-sized cells under different conditions

In conjunction with the panel experiments, we determined the cell motility of small cells under different combinations of induction and starvation. Besides the induction medium, we also added the equivalent amount of trace dSi we measured from the induced well plates (~50 µM) on dSi-starved cells to compare the effect of dSi and induction on motility. After 6 h of light exposure and before bead addition, we took 1 min movies of cells and processed them as

described beforehand in our dP medium exchange experiment. A linear mixed effects (LME) model coupled with the post-hoc Tukey's HSD was used to analyze the speed data (Supplementary Figure S2, Table S8).

Supplementary References

1. Maier I, Calenberg M. Effect of Extracellular Ca^{2+} and Ca^{2+}-Antagonists on the Movement and Chemoorientation of Male Gametes of *Ectocarpus siliculosus* (Phaeophyceae). Bot Acta. 1994;107**:**451-460.

2. Guillard RRL. Culture of phytoplankton for feeding marine invertebrates. In: Smith LH, Chanley MH (eds). *Culture of Marine Invertebrate Animals*. Plenum Press: New York, USA. 1975 pp 26–60.

3. Bondoc KGV, Lembke C, Vyverman W, Pohnert G. Selective chemoattraction of the benthic diatom *Seminavis robusta* to phosphate but not to inorganic nitrogen sources contributes to biofilm structuring. Microbiol Open. 2018; in press.

Supplementary Movie Legends

Supplementary Movie 1. This 10 min movie shows dSi-starved, induced small-sized cells being attracted towards a diproline bead. The video speed was accelerated 50 times, and the scale bar indicates 100 µm. Timestamp denotes min:s.

Supplementary Movie 2. This 10 min movie shows dSi-starved, not induced small-sized cells being attracted towards a dSi bead. We propose that dSi is needed for attraction towards diproline, as starvation of self-inducing cells did not induce diproline attraction. The video speed was accelerated 50 times, and the scale bar indicates 100 µm. Timestamp denotes min:s.

Supplementary Figure Legends

Figure S1. Attraction of *S. robusta* to dSi beads.

(A) After 7 days of starvation, large-sized cells (MT^-, strain 84A) responded to dSi-loaded beads in the same manner as small- and medium-sized cells. An evident accumulation compared to the control was observed (Linear mixed effects modeling, n=3 movies per treatment, p=0.0023). Error bars indicate SEM of the mean and the shaded area shows the LME model fit with 95% confidence intervals. Detailed statistical analysis can be found in Table S6.

(B) Comparison of dSi attraction in large (vegetative), medium (sexual), small-sized (sexual) cells. No statistical difference between all the cell sizes regarding attraction was observed (Linear mixed effects modeling, n=3 movies per treatment for large and medium, n=6 movies per treatment for small p=0.8535). Error bars indicate SEM of the mean and the shaded area shows the LME model fit with 95% confidence intervals. Detailed statistical analysis can be found in Table S7.

Figure S2. Cell speeds of small-sized cells (MT^+ strain 85A) in different combinations of starvation and induction. No difference in motility was observed for non-starved cells, whether induced or not (Linear mixed effects modeling (LME) with Tukey's HSD, p= 0.9999, n=3). On the other hand, dSi-starved and induced cells have significantly lower speed than the not induced counterpart (LME with Tukey's HSD, p<0.0001, n=3). To tease out whether the decreased motility is because of SIP^- or the residual dSi from the induction medium, we added the equivalent dSi measured (~50 µM per well) on dSi-starved cells. Indeed, the residual dSi was sufficient for the slowing down of the cells (LME with Tukey's HSD, p=1.000, n=3). Data points are presented as mean ± SEM of all tracked cells (n=50-175 cells) from three 30-s

movies for each treatment. Bars with same colors are not statistically significant from each other. Detailed statistical analysis can be found in Table S8.

Figure S3. Modeling results using a broad parameter range. Vertical lines on subplots represent thresholds between size classes of diatom individuals that imply a switch between optimal modes of reproduction. The first threshold (transition red-to-brown) appears if the optimization criterion is solely the expected growth-rate (solid line). By adding a second optimization criterion, the estimated offspring size (dashed line), in linear combination with the estimated growth-rate (dotted line) a second threshold was determined (transition brown-to-green). Individuals of size classes within the red area should choose meiosis as the primary mode of reproduction, while individuals of size classes within the green area should always choose mitosis. The brown area represents size classes of individuals that should choose mitosis according to the optimal expected number of offspring but may choose meiosis to additionally preserve mean cell size. The plots show that the optimal switching points significantly alter along typical population dynamics and resource availability. Note that the mean long-term growth rates (GR) given are a fitness proxy and not equivalent to growth rates in cultures. The term *gr_mitosis* stands for mitotic growth rate (proxy for availability of resources) and *r_enc* is detection rate of mate-finding cells (proxy for cell density/SIP availability).

Supplementary Figures

Figure S1

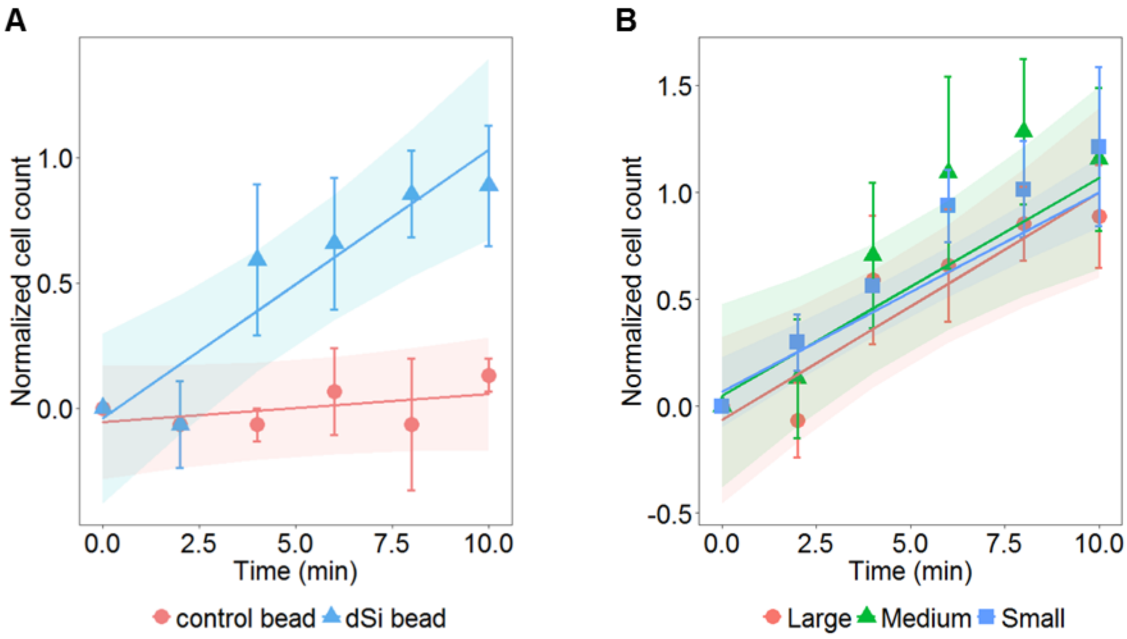

Figure S2

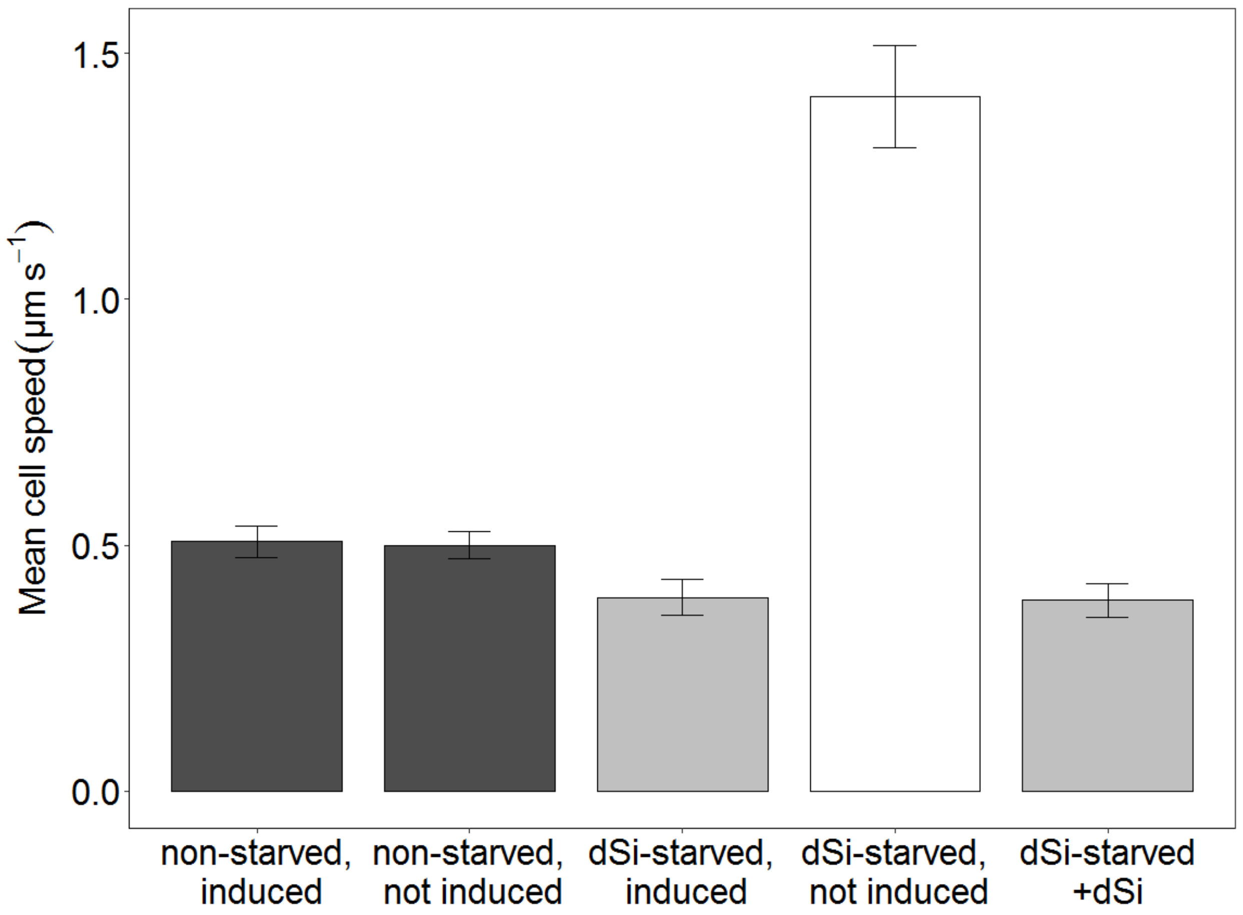
Mean cell speed (μm s−1)
1.5
1.0
0.5
0.0
non-starved, induced
non-starved, not induced
dSi-starved, induced
dSi-starved, not induced
dSi-starved +dSi

Figure S3.

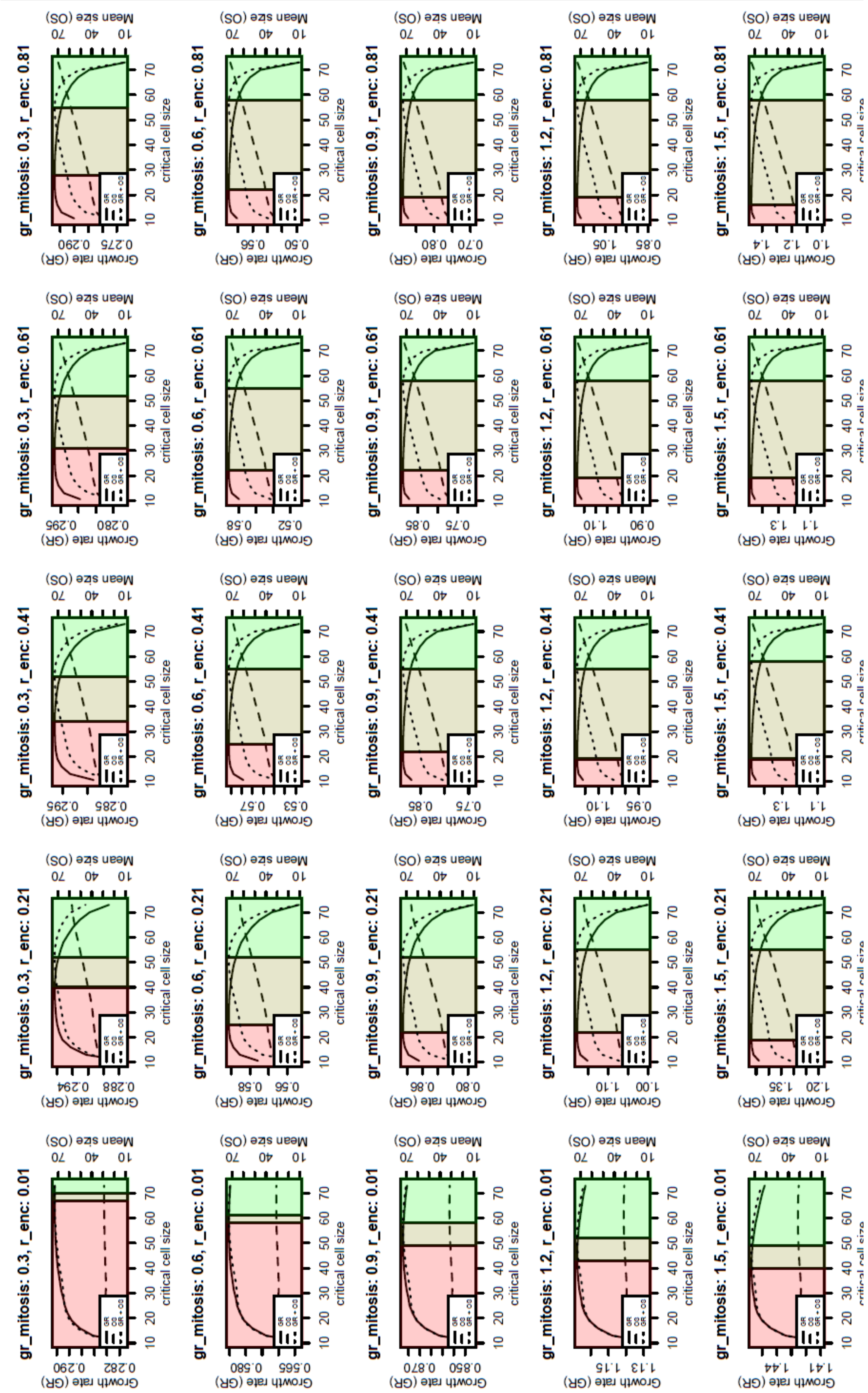

gr_mitosis: 0.3, r_enc: 0.81
gr_mitosis: 0.6, r_enc: 0.81
gr_mitosis: 0.9, r_enc: 0.81
gr_mitosis: 1.2, r_enc: 0.81
gr_mitosis: 1.5, r_enc: 0.81
gr_mitosis: 0.3, r_enc: 0.61
gr_mitosis: 0.6, r_enc: 0.61
gr_mitosis: 0.9, r_enc: 0.61
gr_mitosis: 1.2, r_enc: 0.61
gr_mitosis: 1.5, r_enc: 0.61
gr_mitosis: 0.3, r_enc: 0.41
gr_mitosis: 0.6, r_enc: 0.41
gr_mitosis: 0.9, r_enc: 0.41
gr_mitosis: 1.2, r_enc: 0.41
gr_mitosis: 1.5, r_enc: 0.41
gr_mitosis: 0.3, r_enc: 0.21
gr_mitosis: 0.6, r_enc: 0.21
gr_mitosis: 0.9, r_enc: 0.21
gr_mitosis: 1.2, r_enc: 0.21
gr_mitosis: 1.5, r_enc: 0.21
gr_mitosis: 0.3, r_enc: 0.01
gr_mitosis: 0.6, r_enc: 0.01
gr_mitosis: 0.9, r_enc: 0.01
gr_mitosis: 1.2, r_enc: 0.01
gr_mitosis: 1.5, r_enc: 0.01
critical cell size
Growth rate (GR)
Mean size (OS)
GR
OS
GR+OS

Supplementary Table Legends

Table S1. Detailed statistical parameters for each model fit of the corresponding figures mentioned.

Table S2. Description of the parameters used in the decision-making numerical model.

Table S3. Pair-wise comparisons using Tukey's HSD of cell accumulation around a diproline or a dSi bead on different combinations of dSi and induction medium availability. Individual post-hoc tests were done per bead for both small and medium-sized cells.

Table S4. Linear mixed effects (LME) model results on count data (Z-standard score transformed) on the choice experiment (diproline-loaded bead vs. dSi-loaded bead) for two different treatments (induced and not induced).

Table S5. Pair-wise comparisons using Tukey's HSD of speed (log+1 transformed) around a diproline, dSi, or outside the beads on the choice experiment for two different treatments (induced and not induced).

Table S6. Linear mixed effects (LME) model results on count data (Z-standard score transformed) on the dSi attraction experiment of large-sized cells (strain 84A) (dSi-loaded beads vs. control beads).

Table S7. Linear mixed effects (LME) model results on count data (Z-standard score transformed) on the dSi attraction experiment of large, medium, and small-sized cells.

Table S8. Pair-wise comparisons using Tukey's HSD of cell speeds (log+1 transformed) in different combinations of starvation and induction and with the addition of trace amounts of dSi (~50 µM per well) 6 h after induction.

Supplementary Tables

Table S1. Detailed statistical parameters for each model fit of the corresponding figures mentioned.

Figure	Experimental Set	Supplementary Result Table	Sample Size (n)	Model	Random Factor	Correlation Structure	Variance Structure	Extra processing
1	Medium-sized and small-sized panels	Table S3	$n_{replicates} = 3$; except for 85AS, dSi-starved, induced (diproline bead) which has 6	LME (per bead) with post-hoc test (Tukey's HSD): cellsBase[a] ~ treatment*time	Replicate ID	Replicate ID and treatment	Treatment	-
2a	Choice: cell count	Table S4	$n_{movies} = 5$, $n_{cells/movie} = 80$-100	LME (per treatment): cellsBase[a] ~ bead*time	Replicate ID	Replicate ID and treatment	Bead	Cells in overlapping bins were excluded from analysis
2b	Choice: speed	Table S5		LME (per treatment): Vlog[b] ~ bead*time	Track ID	Track ID and bin	Bead	
Supp 1a	dSi attraction of large cells	Table S6	$n_{movies} = 3$	LME: cellsBase[a] ~ bead*time	Replicate ID	-	Bead	-
Supp 1b	dSi attraction across cell sizes	Table S7	$n_{movies} = 3$ (large cells), 5 (medium and small cells)	LME: cellsBase[a] ~ cell size*time	Replicate ID	Replicate ID and cell size	Replicated ID	
Supp 2	Small-sized cells medium exchange experiment	Table S8	$n_{movies} = 3$, $n_{cells/movie} = 50$-175	LME with post-hoc test (Tukey's HSD): Vlog[b] ~ treatment	Track ID	-	Treatment	1st 30s of video was removed, only complete 30s data were used for analysis

[a]cellsBase = Z standardized cell count calculated per treatment using the formula: $Z = (X-\mu)/\sigma$, where μ is mean, X is score and σ is standard deviation
[b]Vlog = log+1 transformation of speed

Table S2. Description of the parameters used in the decision-making numerical model.

Parameter	Value	Unit	Description
$size_{inital}$	75	µm	Initial size of diatoms
$size_{min}$	10	µm	Minimal size of viable diatoms
$size_{crit}$	variable	µm	Diatoms below this size threshold are susceptible to SIPs and produce SIPs
$gr_{mitosis}$	variable	d^{-1}	Mitotic growth rate
$t_{d_{mit}}$	variable	d^{-1}	Mitotic doubling time (generation time) calculated from mitotic growth rate as: $t_{d_{mit}} = \frac{\ln(2)}{gr_{mitosis}}$
$time_{meiosis}$	1.125	d	Meiotic generation time
$shrinkage$	0.7	$\frac{\mu m}{t_{d_{mit}}}$	Decrease in size during mitotic division
g_{max}	4500		Maximum number of mitotic generations used for fitness estimation
r_{enc}	variable	d^{-1}	Rate at which a diatom individual is able to detect and reach another individual within its detection radius (or neighborhood, influenced by diffusivity / amount / detection efficiency of SIPs)

Table S3. Pair-wise comparisons using Tukey's HSD of cell accumulation around a diproline or a dSi bead on different combinations of dSi and induction medium availability. Individual post-hoc tests were done per bead for both small and medium-sized cells.

Pair-wise comparisons	Estimate	Std. error	z-value	Pr(>|z|)
**Medium-sized cells: diproline bead**				
**starved, induced – starved, not induced**	-0.07962	0.06261	-1.272	0.55117
**starved, induced – non-starved, induced**	-0.83577	0.18414	-4.539	< 1e-04 ***
**starved, not induced – non-starved, not induced**	0.02181	0.13974	0.156	0.99847
**starved, not induced – non-starved, induced**	-0.91539	0.17930	-5.105	< 1e-04 ***
**non-starved, induced – non-starved, not induced**	-0.93720	0.22252	-4.212	0.00012 ***
**Medium-sized cells: dSi bead**				
**starved, induced – starved, not induced**	0.73348	0.20706	3.542	0.00207 **
**starved, induced – non-starved, induced**	-0.01826	0.13018	-0.140	0.99896
**starved, induced – non-starved, not induced**	-0.07387	0.12413	-0.595	0.93044
**starved, not induced – non-starved, not induced**	0.65961	0.22023	2.995	0.01404 *
**starved, not induced – non-starved, induced**	0.71522	0.22370	3.197	0.00694 **
**non-starved, induced – non-starved, not induced**	0.05561	0.15024	0.370	0.98181
**Small-sized cells: diproline bead**				
**starved, induced – starved, not induced**	-0.6705	0.3388	-1.979	0.18072
**starved, induced – non-starved, induced**	-0.6855	0.4914	-1.395	0.47845
**starved, induced – non-starved, not induced**	-0.5879	0.5587	-1.052	0.69845
**starved, not induced – non-starved, not induced**	-1.2585	0.4490	-2.803	0.02409 *
**starved, not induced – non-starved, induced**	-1.3560	0.3617	-3.749	0.00105 **
**non-starved, induced – non-starved, not induced**	-0.0975	0.5729	-0.170	0.99805
**Small-sized cells: dSi bead**				
**starved, induced – starved, not induced**	0.546984	0.149620	3.656	0.00133 **
**starved, induced – non-starved, induced**	0.051255	0.134189	0.382	0.98042
**starved, induced – non-starved, not induced**	0.000513	0.150551	0.003	1.00000
**starved, not induced – non-starved, not induced**	0.547497	0.189955	2.882	0.01972 *
**starved, not induced – non-starved, induced**	0.598240	0.177268	3.375	0.00379 **
**non-starved, induced – non-starved, not induced**	0.050742	0.178055	0.285	0.99167

Signif. codes: 0 '***' 0.001 '**' 0.01 '*' 0.05 '.' 0.1 ' ' 1 (Adjusted p values reported -- single-step method)

Table S4. Linear mixed effects (LME) model results on count data (Z-standard score transformed) on the choice experiment (diproline-loaded bead vs. dSi-loaded bead) for two different treatments (induced and not induced).

	numDF	denDF	F-value	p-value
**Induced**				
**(intercept)**	1	48	2.170292	0.1472
**bead**	1	8	14.231266	0.0054
**time**	1	48	1.902299	0.1742
**bead:time**	1	48	5.282655	0.0259
**Not induced**				
**(intercept)**	1	48	15.13610	0.0003
**bead**	1	8	14.01567	0.0057
**time**	1	48	19.14796	0.0001
**bead:time**	1	48	15.38768	0.0003

Table S5. Pair-wise comparisons using Tukey's HSD of speed (log+1 transformed) around a diproline, dSi, or outside the beads on the choice experiment for two different treatments (induced and not induced)

Pair-wise comparisons	Estimate	Std. error	z-value	Pr(>|z|)
**Induced**				
**diproline – dSi**	-0.21276	0.03375	-6.305	<0.001 ***
**diproline – outside beads**	-0.15616	0.02216	-7.048	<0.001 ***
**dSi – outside beads**	0.05660	0.02758	2.053	0.0953 .
**Not induced**				
**diproline – dSi**	0.14404	0.03555	4.052	0.000144 ***
**diproline – outside beads**	-0.02178	0.03192	-0.682	0.764509
**dSi – outside beads**	-0.16581	0.01845	-8.989	< 1e-05 ***

Signif. codes: 0 '***' 0.001 '**' 0.01 '*' 0.05 '.' 0.1 ' ' 1 (Adjusted p values reported -- single-step method)

Table S6. Linear mixed effects (LME) model results on count data (Z-standard score transformed) on the dSi attraction experiment of large-sized cells (strain 84A) (dSi-loaded beads vs. control beads).

	numDF	denDF	F-value	p-value
**(intercept)**	1	27	5.515138	0.0264
**bead**	1	4	8.616357	0.0426
**time**	1	27	6.801745	0.0147
**bead:time**	1	27	11.34181	0.0023

Table S7. Linear mixed effects (LME) model results on count data (Z-standard score transformed) on the dSi attraction experiment of large, medium, and small-sized cells.

times	numDF	denDF	F-value	p-value
**(intercept)**	1	56	108.69821	<.0001
**size**	2	9	0.16117	0.8535
**time**	1	56	82.95899	<.0001
**size:time**	2	56	0.10371	0.9017

Table S8. Pair-wise comparisons using Tukey's HSD of cell speeds (log+1 transformed) in different combinations of starvation and induction and with the addition of trace amounts of dSi (~50 µM per well) 6 h after induction.

Pair-wise comparisons	Estimate	Std. error	z-value	Pr(>|z|)
**not starved, not induced – starved, induced**	0.0016413	0.0196737	0.083	0.99999
**starved+dSi – not starved, induced**	-0.0733831	0.0208220	-3.524	0.00342 **
**starved, induced – not starved, induced**	-0.0726083	0.0224860	-3.229	0.00976 **
**starved, not induced – not starved, induced**	0.3742123	0.0443702	8.434	< 0.001 ***
**starved+dSi – not starved, not induced**	-0.0750244	0.0206693	-3.630	0.00245 **
**starved, induced – not starved, not induced**	-0.0742497	0.0223446	-3.323	0.00718 **
**starved, not induced – not starved, not induced**	0.3725710	0.0442987	8.410	< 0.001 ***
**starved, induced – starved+dSi**	0.0007748	0.0233620	0.033	1.00000
**starved, not induced –starved+dSi**	0.4475954	0.0448205	9.986	< 0.001 ***

Signif. codes: 0 '***' 0.001 '**' 0.01 '*' 0.05 '.' 0.1 ' ' 1 (Adjusted p values reported -- single-step method)
